# Underuse of statins in MASLD despite population-based associations with lower liver stiffness

**DOI:** 10.1016/j.jhepr.2026.101764

**Published:** 2026-02-11

**Authors:** Jesse Pustjens, Laurens A. van Kleef, Jelena Pavlović, Lies Lahousse, Adriaan G. Holleboom, Harry L.A. Janssen, Ibrahim Ayada, Maryam Kavousi, Layal Chaker, Jeanine E. Roeters van Lennep, Robert J. De Knegt, Bettina E. Hansen, Bruno H. Stricker, Maarten J.G. Leening, Willem Pieter Brouwer

**Affiliations:** 1 Department of Gastroenterology and Hepatology, Erasmus MC - University Medical Center Rotterdam, Rotterdam, The Netherlands; 2 Department of Epidemiology, Erasmus MC - University Medical Center Rotterdam, Rotterdam, The Netherlands; 3 Department of Internal and Vascular Medicine, Amsterdam University Medical Centres, Amsterdam, The Netherlands; 4 Toronto Centre for Liver Disease, Toronto General Hospital, University Health Network, Toronto, Canada; 5 Department of Internal Medicine, Erasmus MC- University Medical Center Rotterdam, Rotterdam, The Netherlands; 6 IHPME, University of Toronto, Toronto, Canada; 7 Department of Cardiology, Erasmus MC - University Medical Center Rotterdam, Rotterdam, The Netherlands; 8 Department of Radiology, Erasmus MC - University Medical Center Rotterdam, Rotterdam, The Netherlands

**Keywords:** MASLD, fibrosis, liver stiffness, statin, epidemiology, population based

## Abstract

**Background & Aims:**

Statins, used for cardiovascular disease (CVD) prevention, may offer hepatoprotective benefits. However, adherence to treatment indications and the associations between statin use, metabolic dysfunction-associated steatotic liver disease (MASLD), and elevated liver stiffness in the general population remain poorly understood.

**Methods:**

This prospective, population-based study included adults aged ≥40 years between 2011 and 2020. We evaluated statin indications for CVD risk using prevailing European Society of Cardiology/European Atherosclerosis Society clinical practice guidelines based on SCORE2 and SCORE2-Older Persons algorithms. We used multivariable regression to examine associations between statins, MASLD, and elevated liver stiffness, adjusting for demographic, socioeconomic and metabolic covariables. We performed dose-response analyses using WHO defined daily dosages.

**Results:**

Of 6,405 eligible individuals, 6,055 participants were included in the analysis (median age 64 years; 56% female); MASLD was present in 32%, elevated liver stiffness in 4.8%, and statin use in 21%. Participants with MASLD had higher predicted 10-year CVD risk compared to participants without MASLD (*p <*0.001), yet were less likely to use statins: 33% of individuals with MASLD and an indication for statin treatment remained untreated, compared to 19% of those without MASLD (*p <*0.001). Statin use was associated with lower prevalence of MASLD (adjusted odds ratio 0.76; 95% CI 0.63–0.92) and elevated liver stiffness (adjusted odds ratio 0.65; 95% CI 0.46–0.92) relative to untreated individuals with a statin treatment indication. The highest statin WHO defined daily dosage category was associated with lower prevalence of MASLD (*p* = 0.033) and elevated liver stiffness (*p* = 0.035).

**Conclusions:**

Individuals with MASLD are less likely to use statins despite a contemporary guideline-based indication. Statin use is independently associated with lower prevalence of both MASLD and elevated liver stiffness. These findings underscore the need to improve CVD risk management in this population with the potential added benefit of mitigating MASLD.

**Impact and implications:**

In this large prospective, population-based study, statins were underutilized in metabolic dysfunction-associated steatotic liver disease (MASLD) compared to non-MASLD individuals, even though they had the highest cardiovascular risk and met guideline-based treatment criteria. Our findings further demonstrate that individuals who used statins had a lower likelihood of MASLD and elevated liver stiffness compared with statin-eligible individuals who were not treated. Taken together, these results highlight a missed opportunity: optimizing statin use in people with MASLD could strengthen cardiovascular disease prevention while also offering potential benefits for liver health.

## Highlights

• Statins are disproportionally underused in the MASLD population.
• Statin use is associated with a lower risk of both MASLD and elevated liver stiffness in the general population.
• These findings highlight an opportunity for clinicians to optimize statin therapy in patients with MASLD.
• This has the potential to not only reduce cardiovascular morbidity and mortality, but also to mitigate MASLD progression.

## Graphical abstract

**Figure:**
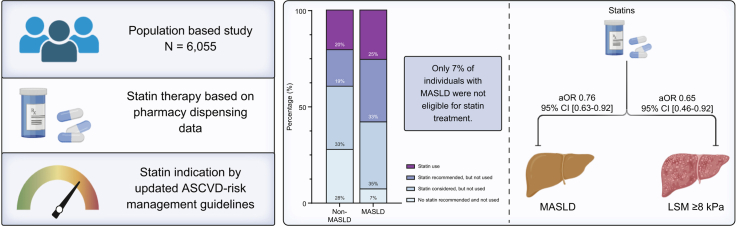

## Introduction

The increasing metabolic burden in the general population has led to a substantial rise in the prevalence of metabolic dysfunction-associated steatotic liver disease (MASLD).^1^^,^^2^ MASLD can be considered the hepatic manifestation of the metabolic syndrome and ranges from a mostly benign disease with simple steatosis to steatohepatitis (MASH) which may progress to cirrhosis and hepatocellular carcinoma.^3^ Despite the increased risk of advanced liver disease, cardiovascular disease (CVD) remains the leading cause of death among individuals with MASLD.^4^

Since MASLD and CVD share common pathophysiological mechanisms and risk factors, there is growing interest in repurposing established cardiovascular therapies for this population.^5^^,^^6^ Statins have been implicated in reducing the risk of hepatic decompensation, portal hypertension, and mortality in patients with advanced liver disease.^7^ In a population-based setting, statins have been associated with lower risk of liver-related events in patients with MASLD.^8^ Prevailing clinical practice guidelines endorse the use of statins in adults with chronic liver disease when indicated for CVD risk reduction.^9^ Since there are no ongoing large randomized controlled trials with histological endpoints evaluating the efficacy of statins in MASLD or MASH, it is unlikely that forthcoming guidelines will incorporate MASLD or MASH as standalone indications for statin therapy in the near-future. However, current CVD risk management guidelines may provide an important therapeutic window for individuals with MASLD, with the primary goal of preventing CVD, but with a potential secondary benefit of mitigating liver disease progression.

Notably, statins appear to be underprescribed: in a study on 255 patients with MASLD and dyslipidemia, only 60% received indicated statin therapy as assessed by the (now outdated) 2004 Adult Treatment Panel III guidelines.^10^^,^^11^ The updated 2025 European Society of Cardiology (ESC) and European Atherosclerosis Society (EAS) management guidelines recommend statin therapy based on individual risk assessment using clinical parameters and risk scores to estimate 10-year composite fatal and non-fatal CVD risk.^12^ The proportion of patients with MASLD meeting treatment eligibility under these current guidelines remains unknown.

In this study, we assess the CVD risk profiles of patients with MASLD and investigate statin prescription rates based on the recently updated 2025 ESC/EAS guidelines. Additionally, we examine the association between statin use, MASLD, and liver stiffness, including potential dose-dependent associations.

## Patients and methods

### Study design and population

This prospective, population-based cohort study is embedded in the Rotterdam Study, which includes inhabitants from the Ommoord district in the city of Rotterdam, the Netherlands. A detailed description of the rationale and data collection for the Rotterdam Study have been reported elsewhere.^13^ Inclusion was based on age (≥40 years) and ZIP code. Participants with unreliable liver stiffness measurements (LSM), viral hepatitis, excessive alcohol consumption (≥20/30 g/day for women/men) or known heart failure were excluded from the analyses.

### Hepatologic assessment

Hepatologic assessment consisted of hepatic ultrasound and LSM measured by vibration-controlled transient elastography (FibroScan, Paris, France). Hepatic steatosis was diagnosed by ultrasound based on increased echogenicity of the liver parenchyma relative to the renal cortex or spleen. MASLD was defined according to the updated Delphi consensus statement as hepatic steatosis in combination with at least one cardiometabolic risk factor (*i.e.* overweight, hypertension, low-HDL, high triglycerides, insulin resistance).^14^ LSM was considered reliable if at least 10 valid measurements were obtained with an IQR ≤30% of the median, for LSM values exceeding 7.0 kPa.^15^ LSM ≥8.0 kPa was considered elevated and a proxy of liver fibrosis.^16^

### Statin treatment, treatment recommendations and dosages

Data on statin prescriptions were obtained from direct linkage with digital pharmacy records, containing dispensing information. If pharmacy data were unavailable, self-reported medication use, collected during a structured home interview, was used (direct pharmacy linkage 70% *vs.* self-reported in 30%). We considered participants to be on statin therapy if they had received treatment for at least the past month prior to the interview.

We defined statin treatment recommendations in accordance with the prevailing 2025 ESC/EAS Guidelines on Cardiovascular Disease Prevention in Clinical Practice.^12^ We used the Systematic Coronary Risk Evaluation 2 (SCORE2) and SCORE2-Older Persons (SCORE2-OP, for individuals aged ≥70 years) algorithms for low-risk countries to estimate 10-year cardiovascular risk, which factor in age, sex, smoking status, total cholesterol, systolic blood pressure, and regional risk classification.^17^ SCORE2 was derived from data of 45 European cohort studies including 677,684 individuals and 30,121 cardiovascular events, with extensive external validation across populations. Sex-specific, competing risk–adjusted Cox proportional hazards models were used to estimate the 10-year risk of first-onset fatal and non-fatal cardiovascular events. The models were recalibrated for four predefined European risk regions to account for regional differences in cardiovascular event rates and risk factor distributions.

Based on age-specific thresholds, participants were categorized into one of three cardiovascular risk groups: (1) low-to-moderate, (2) high, or (3) very high risk (Table 1). Those with a medical history of atherosclerotic CVD, eGFR <30 ml/min, or diabetes and eGFR <45 ml/min were categorized into the very high-risk group.

**Table 1:** Population characteristics.

	Statin-naïve n = 4,747	Statin users n = 1,308
Age (years)	64 (10)	65 (10)
Female, n (%)	2,727 (57)	639 (49)
Household income		
Low	1,100 (25)	350 (27)
Intermediate	1,592 (34)	482 (37)
High	1,686 (35)	387 (30)
Educational attainment		
Primary	297 (6.4)	110 (8.6)
Lower/intermediate general education	1,573 (34)	452 (35)
Higher general education	1,501 (32)	383 (30)
University	1,294 (28)	335 (26)
Current smoking, n (%)	976 (21)	306 (23)
Alcohol intake, grams/day	6.4 [0.5–8.6]	5.4 [0.5–8.6]
BMI, kg/m^2^	27 (4.1)	28 (3.9)
BMI category, n (%)		
Lean	1,596 (34)	341 (26)
Overweight	2,209 (47)	652 (50)
Obese	942 (20)	315 (24)
Hypertension, n (%)	2,739 (58)	829 (63)
Diabetes, n (%)	457 (9.6)	402 (31)
AST (U/L)	23 [20–28]	24 [21–29]
ALT (U/L)	19 [15–26]	21 [16–28]
Total cholesterol (mmol/L)	5.6 [4.9–6.3]	4.8 [4.1–5.5]
LDL-C (mmol/L)	3.5 [2.8–4.1]	2.6 [2.0–3.2]
HDL-C (mmol/L)	1.5 [1.2–1.8]	1.4 [1.1–1.7]
Triglycerides (mmol/L)	1.2 [0.9–1.7]	1.4 [1.0–1.9]
LSM (kPa)	4.5 [3.7–5.6]	4.7 [3.8–5.8]
LSM ≥8 kPa	213 (4.5)	64 (4.9)
MASLD, n (%)	1,469 (31)	490 (38)

Continuous data is presented as median [P25–P75].

ALT, alanine aminotransferase; AST, aspartate aminotransferase; HDL-C, high-density lipoprotein cholesterol; LDL-C low-density lipoprotein-cholesterol; LSM, liver stiffness measurement; MASLD, metabolic dysfunction-associated steatotic liver disease.

Subsequently, in the statin-naïve population, three statin treatment recommendation categories were made, based on predicted risk and the presence of risk-modifying factors, diabetes and estimated kidney function: (1) lifestyle advise, (2) treatment considered, and (3) treatment recommended (Fig. S1).

Statin dose was standardized using the World Health Organization’s Defined Daily Dose (WHO-DDD) methodology, which allows for comparison across different statin types and potencies. The DDD reflects the assumed average maintenance dose per day for a drug used in its main indication in adults.

### Cardiometabolic risk factors and other covariables

An adjudicated medical history of CVD was determined through medical records and included prior myocardial infarction, stroke, transient ischemic attack, carotid endarterectomy, percutaneous coronary intervention, coronary artery bypass grafting, abdominal aortic aneurysm surgery, or peripheral arterial disease. A family history of premature CVD was defined as a self-reported myocardial infarction or stroke in a first-degree relative before the age of 65.

Anthropometrics and blood pressure were measured during standardized examinations conducted by a trained research assistant. Fasting laboratory measurements included, among others, aspartate aminotransferase, alanine aminotransferase, serum glucose, triglycerides, total cholesterol, and high-density lipoprotein cholesterol, using validated protocols. Low-density lipoprotein cholesterol was estimated using the Friedewald equation.

### Statistical analysis

We conducted multivariable logistic regression analyses to assess the association between statin use and the presence of MASLD or elevated liver stiffness. Statin treatment status was categorized into three mutually exclusive groups based on contemporary guideline-recommended indications and current use: (1) statin recommended but not currently using statins (reference group), (2) currently on statin therapy, and (3) statin not recommended and not using. A fourth theoretical category – statin use without indication – was not included, as lipid measurements prior to statin initiation were unavailable.

All models were adjusted for potential confounders selected beforehand based on known associations with our outcomes and cardiovascular risk: age, sex, household income, educational attainment, diabetes, waist circumference, current smoking, and alcohol consumption (grams per day).

We converted statin dosages to WHO-DDDs based on the WHO Anatomical Therapeutic Chemical classification system and categorized these into tertiles to evaluate potential dose-dependent associations with MASLD and liver stiffness.^18^

We conducted a sensitivity analysis in which the statin “treatment considered” group was analyzed separately rather than being grouped with the “not recommended” category.

We used IBM SPSS Statistics, version 28.0.1.0, for all statistical analyses. We considered a two-sided *p* value < 0.05 statistically significant.

### Ethics

The Rotterdam Study was approved by the Medical Ethics Committee of the Erasmus MC (registration number MEC 02.1015) and by the Dutch Ministry of Health, Welfare and Sport (Population Screening Act WBO, license number 1071272-159521-PG). The Rotterdam Study is registered in the WHO International Clinical Trials Registry Platform. All participants provided written informed consent for participation and for the collection of information from their treating physicians.

## Results

### Population

In the base cohort, 6,405 participants were initially eligible for inclusion. After excluding 350 individuals due to unreliable LSM, excessive alcohol intake, known viral hepatitis, or a history of heart failure, 6,055 participants remained in the study. The median age was 64 years [IQR 56–71], 56% were female, and 14% had diabetes. Baseline characteristics of the study population, stratified by statin use, are presented in Table 1.

MASLD was present in 32% of participants (n = 1,959), while elevated liver stiffness was observed in 4.6% (n = 277). Overall, 21% (n = 1,308) of participants were using statins. Among statin users, the median WHO-DDD was 1.0 [IQR 0.67–1.33], and the median duration of use was 5.9 years [IQR 3.0–10.0].

### Cardiovascular risk

A history of CVD was present in 4.8% (n = 292) of the total population, equally distributed among individuals with and without MASLD (4.8% *vs.* 4.8%). However, based on age-specific SCORE2 and SCORE2-OP risk assessments, participants with MASLD exhibited substantially higher predicted 10-year CVD risk (Fig. S1): Among statin-naïve participants without MASLD, 54% were classified as having low-to-moderate CVD risk, compared to only 37% of those with MASLD (*p <*0.001). Conversely, 21% of participants with MASLD were categorized as very high risk, compared to 13% participants without (*p <*0.001).

**Fig. 1:**
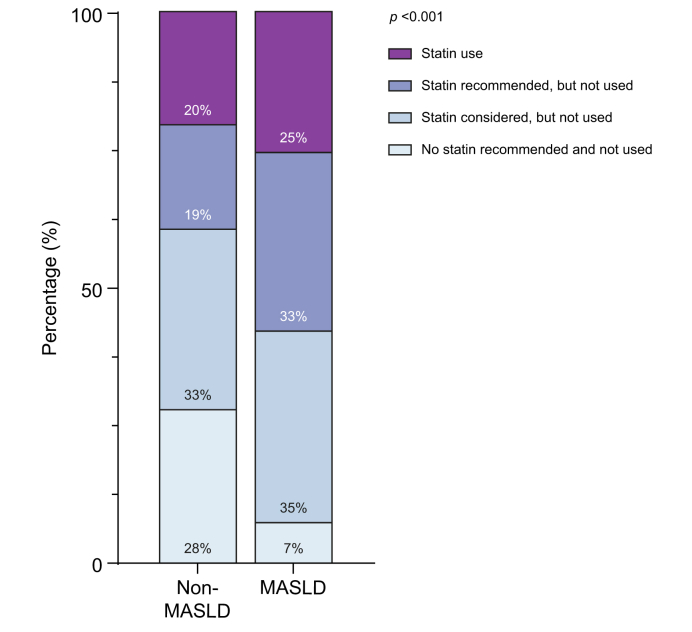
Contemporary statin indication and treatment status among participants with and without MASLD. Bars represent the distribution of statin eligibility categories according to 2025 ESC/EAS guidelines on cardiovascular disease prevention. Results obtained using the chi-square test. ESC/EAS, European Society of Cardiology/European Atherosclerosis Society; MASLD, metabolic dysfunction-associated steatotic liver disease.

Statin use was more common among individuals with MASLD compared to those without (25% *vs.* 20%; *p <*0.001). However, a greater proportion of MASLD participants met contemporary guideline-recommended indications for statin therapy while they were not treated (Fig. 1). Specifically, one in three (n = 1,316/4,017) participants with MASLD was not treated with a contemporary treatment indication, compared to one in five (n = 626/1928) participants without MASLD (*p <*0.001). Moreover, only 7% (n = 142/1928) of participants with MASLD had no contemporary indication for statins *vs*. 28% (n = 1,121/4,017) of those without MASLD (*p* >0.001).

### MASLD and elevated liver stiffness

**Fig. 2:**
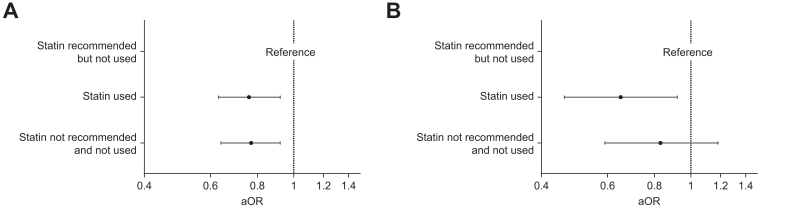
Association of statin use with MASLD and elevated liver stiffness according to ESC/EAS guideline recommendations. Results were obtained with logistic regression models and presented as aORs with 95% CIs. Models are adjusted for age, sex, diabetes, waist circumference, current smoking, alcohol consumption (grams per day), household income (tertiles) and educational attainment. aORs, adjusted odds ratios; ESC/EAS, European Society of Cardiology/European Atherosclerosis Society; MASLD, metabolic dysfunction-associated steatotic liver disease.

In multivariable logistic regression models adjusted for demographic, socioeconomic, and cardiometabolic confounders, participants using statins had a significantly lower prevalence of MASLD than participants with a treatment indication who were not receiving statins (adjusted odds ratio [aOR] 0.76, 95% CI 0.63–0.92) (Fig. 2). A similar pattern was observed among those without a statin treatment indication (aOR 0.77, 95% CI 0.64–0.92).

In those with an indication, statin therapy was associated with a significantly lower prevalence of elevated liver stiffness (aOR 0.65, 95% CI 0.46–0.92) compared to no treatment. No significant association was observed among those without a statin indication (aOR 0.83, 95% CI 0.59–1.18). Similar effect sizes were observed whether statin treatment was defined through direct pharmacy linkage or self-reported via questionnaires.

Among participants not using statins but for whom treatment could be considered, the prevalence of MASLD was lower than in participants with a guideline-based indication who were not receiving statins (aOR 0.80, 95% CI 0.67–0.96) and similar to that of statin users (aOR 0.74, 95% CI 0.61–0.89) (Table S2).

### Treatment intensity

**Fig. 3:**
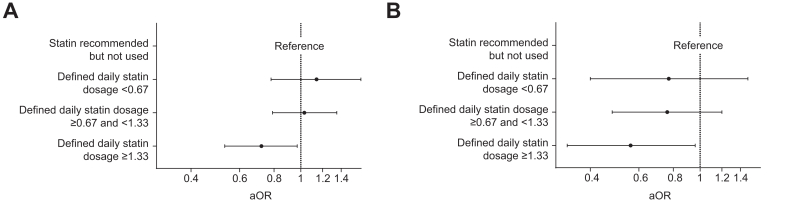
Likelihood of MASLD and elevated liver stiffness associated with defined daily statin dosage tertiles. Results were obtained with logistic regression models and presented as aORs with 95% CIs. Models are adjusted for age, sex, diabetes, waist circumference, current smoking, alcohol consumption (grams per day), household income (tertiles) and educational attainment. aORs, adjusted odds ratios; ESC/EAS, European Society of Cardiology/European Atherosclerosis Society; MASLD, metabolic dysfunction-associated steatotic liver disease.

WHO-DDD was available through pharmacy linkage in 70% (n = 4,243) of all participants. We found a dose–response relation between the WHO-DDD of statin use and the presence of both MASLD and elevated liver stiffness (Fig. 3). The highest tertile was significantly associated with a lower prevalence of both MASLD (aOR 0.70, 95% CI 0.56–0.97) and elevated liver stiffness (aOR 0.59, 95% CI 0.35–0.98) in multivariable analyses.

## Discussion

In this prospective, population-based cohort study of 6,055 participants, we observed that one in three individuals with MASLD were not receiving appropriate statin therapy as recommended by prevailing clinical guidelines compared to just one in five among those without MASLD. This disproportionately low use is concerning, not only from a cardiovascular risk management perspective but also potentially from a hepatologic standpoint, as observational data suggest that statin use is associated with a significantly lower prevalence of both MASLD and elevated liver stiffness.

Alongside lifestyle modification, statins are a cornerstone of cardiovascular disease prevention. In individuals with MASLD, among whom CVD is the leading cause of death, preventive strategies should be prioritized.^4^ Nevertheless, our data demonstrate that there may be significant untapped preventive potential among individuals with MASLD: one-third did not use statins while meeting contemporary criteria for treatment, and another third did not use statins even though statin use could be considered according to contemporary 2025 ESC/EAS guidelines. Prior studies have reported statin underuse in MASLD populations, although these studies were limited by use of highly selected populations, smaller sample sizes and older guideline recommendations concerning treatment indications. In a cohort of 605 patients referred for screening for suspected metabolic diseases, 66% of participants with MASLD were not using a statin despite qualifying for one based on guideline recommendations.^19^ Similarly, among 255 veterans with MASLD and dyslipidemia, 40% were not using a statin despite being eligible based on current guideline recommendations.^10^ Lower statin use among individuals with MASLD may, to some extent, be explained by lingering concerns about the hepatotoxicity of statins, leading some clinicians to withhold or deprescribe statins when treating patients with MASLD. However, randomized clinical trial evidence indicates that statin treatment is safe and that elevated transaminases should not be a barrier to prescribing statins.^20^^,^^21^

In our study, individuals who used a statin had a 24% lower prevalence of MASLD compared to those not using a statin in the presence of contemporary guideline recommendations, with more pronounced associations observed among those using higher statin dosages. Additionally, statin users had a 35% lower prevalence of elevated liver stiffness compared to individuals who met the indication for treatment but were not using statins. These findings also align with prior clinical evidence demonstrating that statin use in patients with MASLD was linked to a reduced risk of liver-related events and slower LSM progression.^5^ Likewise, in a historical cohort study of over 16,000 adults with chronic liver disease, statin use was significantly associated with a lower 10-year cumulative incidence of hepatocellular carcinoma.^22^

Our study extends these findings by demonstrating that these associations are generalizable to the community-dwelling population. Notably, statin users in our cohort had about the same risks as those without a treatment indication. These results are reassuring, especially since lifestyle interventions are notoriously difficult to adhere to and often yield modest results.^23^

In a population burdened with a rising prevalence of chronic liver disease, but with limited available treatment options, statins may be a promising, safe, and low-cost treatment, which most clinicians are already highly familiar with. Although MASLD guidelines do not recommend presence of MASLD as a standalone indication for statin initiation due to the lack of randomized clinical trials with histological endpoints, our study highlights an important clinical opportunity: under prevailing CVD prevention guidelines, an additional two-thirds of individuals with MASLD are eligible for statin treatment on top of the one-in-four who already use statins. This provides an important window for clinicians to improve CVD risk management while also leveraging possible hepatoprotective benefits.

Our study was conducted in a well-defined, population-based cohort with extensive data that allowed us to accurately calculate cardiovascular risk based on prevailing ESC/EAS guidelines. Another strength is the availability of statin prescription data through direct pharmacy linkage in 70% of cases, thereby minimizing misclassification by self-reporting. Nonetheless, some limitations should be considered. First, although we employed multivariable adjustment for a number of known potential confounders, residual confounding remains a possibility. Therefore, due to the observational design of the study, causality of statin use with liver outcomes cannot be established. Randomized clinical trials are needed to determine whether the observed associations translate into actionable clinical improvement in hepatic outcomes and, if so, whether they are solely lipid-mediated or reflect potential pleiotropic effects of statins. Second, while dispensing records confirm prescription pick-up, actual statin intake cannot be guaranteed, potentially leading to misclassification and an underestimation of the observed associations. Additionally, the reason for non-use is not known and may involve both physician-related and patient-related factors. Third, due to the population-based design of our study, histological data were not available, as liver biopsy is not feasible in this setting. However, LSM is a well-established and validated non-invasive alternative to assess liver fibrosis.^24^ Nonetheless, severe hepatic steatosis may lead to a higher rate of false positives. The impact of this potential bias is likely limited, as the appropriate use of the XL probe in this study enhances measurement reliability in individuals with higher BMI.^25^ Furthermore, B-mode ultrasonography was used to assess hepatic steatosis, which has limited sensitivity for detecting mild steatosis and is more susceptible to inter- and intra-observer variability compared with more objective measures such as the controlled attenuation parameter.^26^^,^^27^ Consequently, some individuals with mild steatosis may have been misclassified as non-MASLD. Such misclassification would likely bias our findings toward the null, leading to an underestimation of the associations between statin use and MASLD.

We demonstrate that most individuals with MASLD are at very high predicted cardiovascular risk and the majority do not use statins. Statin use was associated with a significantly lower prevalence of both MASLD and elevated liver stiffness with evidence of dose dependency. Our findings highlight an opportunity for clinicians to optimize statin therapy in patients with MASLD, not only to reduce cardiovascular morbidity and mortality, but potentially to mitigate MASLD progression.

## Abbreviations

aOR, adjusted odds ratio; CVD, cardiovascular disease; DDD, defined daily dose; EAS, European Atherosclerosis Society; ESC, European Society of Cardiology; LSM, liver stiffness measurement; MASLD, metabolic dysfunction-associated steatotic liver disease; MASH, metabolic dysfunction-associated steatohepatitis; SCORE2, Systematic Coronary Risk Evaluation 2; SCORE2-OP, SCORE2 for Older Persons.

## Authors’ contributions

Collection of data: JP, LvK, WPB, BHS, JP; Study design, data analysis, writing of the manuscript: JP; LvK, WPB; Critical review of the manuscript, writing of the manuscript, approval of final version and approval of submission: all authors.

## Data availability

Data from the Rotterdam Study can be obtained on request. Requests should be directed toward the management team of the Rotterdam Study (secretariat.epi@erasmusmc.nl), which has a protocol for approving data requests. Because of restrictions based on privacy regulations and informed consent of the participants, data cannot be made freely available in a public repository.

## Financial support

Financial support was provided by the 10.13039/501100015383Foundation for Liver and Gastrointestinal Research, Rotterdam, the Netherlands.

The funding source did not influence the study design, data collection, analysis and interpretation of the data, nor the writing of the report and decision to submit for publication.

## Conflicts of interest

J.P. Nothing to disclose. L.v.K. Nothing to disclose. J.P. Nothing to disclose. L.L. Has been consulted as expert for AstraZeneca, GlaxoSmithKline and Sanofi, and has given lectures sponsored by Chiesi, Johnson and Johnson, IPSA vzw and Domus Medica vzw (non-profit organizations facilitating lifelong learning for health care providers), all paid to her institution. A.G.H. Received research grants from Gilead and Novo Nordisk, acted as consultant for Gilead, Echosens, Novo Nordisk, Norgine, Julius Clinical, Inventiva and Boehringer Ingelheim, (co)leads the LEGEND trial with Inventiva and the SYNCH trial with Caelus Health and the Akkermansia Company and participates in trials of 89BIO, Boehringer Ingelheim, Novo Nordisk and Inventiva. H.L.A.J Received grants from Gilead Sciences, GSK, Janssen, Roche and Vir Biotechnology inc, and is a consultant for Aligos, Gilead sciences, GSK, Janssen, Roche, Vir Biotechnology inc. and Precision Biosciences. I.A. Nothing to disclose. M.K. Nothing to disclose. L.C. Nothing to disclose. J.R.v.L. Received an investigator initiated grant from Novartis paid to department. R.J.d.K. Has contracted research with Echosens, Gilead Sciences, Inventiva Pharma and Janssen, and is advisor for Bracoo and Echosens. B.E.H. Received grants from Ipsen, grants from Gilead, grants from Mirum, personal fees from Ipsen Advisor, personal fees from Gilead Advisor, personal fees from Mirum Advisor, personal fees from Pliant Advisor, personal fees from Advanz Advisor, and personal fees from Intercept Advisor outside the submitted work. B.H.S. Nothing to disclose. M.J.G.L. Received speaker fees from Amgen, Daiichi Sankyo, Novartis, Novo Nordisk, and Sanofi; served as a consultant for Boehringer Ingelheim, Daiichi Sankyo, MSD, Novartis, and Sanofi; received unrestricted educational grant paid to institution from Amarin; and received unrestricted research grants paid to institution from the Netherlands Organisation for Health Research and Development (ZonMw), the Netherlands Organisation for Scientific Research (NWO), the Ministry of Health, Welfare and Sport, the Dutch Heart Foundation, Erasmus MC – University Medical Center Rotterdam, the Diabetes Fonds, Amgen, AstraZeneca, Novartis, Novo Nordisk, and Sanofi. W.P.B. Received speaker fees from Eli Lilly, is part of the advisory board of Novo Nordisk and participates in trials of 89BIO, Boehringer Ingelheim, Novo Nordisk, Inventiva Pharma.

Please refer to the accompanying ICMJE disclosure forms for further details.
