# Supplementary material for: Underuse of statins in MASLD despite population-based associations with lower liver stiffness
Source: JHEP Rep. 2026 Feb 11;8(4):101764. doi: 10.1016/j.jhepr.2026.101764 (PMC13019549; doi:10.1016/j.jhepr.2026.101764)
Supplement: Multimedia component 1 [file mmc1.pdf]

Underuse of statins in MASLD despite population-based associations with lower liver stiffness

Jesse Pustjens, Laurens A. van Kleef, Jelena Pavlović, Lies Lahousse, Adriaan G. Holleboom, Harry L.A. Janssen, Ibrahim Ayada, Maryam Kavousi, Layal Chaker, Jeanine E. Roeters van Lennep, Robert J. De Knegt, Bettina E. Hansen, Bruno H. Stricker, Maarten J.G. Leening, Willem Pieter Brouwer

Table of contents

Fig. S1……………………………………………………………………………….…...2

Table S1……………………………………………………………………………….3

Fig. S2………………………………………………………………………….……...4

Table S2……………………………………………………………………………….5

Recommendation	Age (y)	10-year total ASCVD risk (SCORE2/2-OP), and/ or risk modifying factors
Treatment recommended	-	eGFR <60 ml/min/1.73m^2 if no dialysis
	<50	≥7.5%; or ≥2.5% and DM
	50 to 70	≥10%; or ≥5 to 10% and ≥1 RMF; or ≥5% and DM
	≥70	≥15%; or ≥7.5% and DM
Treatment considered	<50	≥2.5 to 7.5%; or <2.5% and ≥1 RMF
	50 to 70	≥5 to 10% and no RMF; or <5% and ≥1 RMF
	≥70	≥7.5 to 15%; or <7.5% and ≥1 RMF
Lifestyle advise only	<50	<2.5% and no RMF and no DM
	50 to 70	<5% and no RMF and no DM
	≥70	<7.5% and no RMF and no DM

Fig. S1. Treatment recommendation categories following the 2025 ESC/EAS guidelines for the management of dyslipidaemias

SCORE2 was used to calculate predicted ASCVD risk in adults <70 years, and SCORE2-OP in adults ≥70 years of age. Risk modifying factors (RMF) include triglycerides >2.3 mmol/L (200 mg/dL); ankle brachial index <0.9; waist circumference >88 cm in women and >102 cm in men; family history of premature ASCVD.

Abbreviations: ASCVD, atherosclerotic cardiovascular disease; eGFR, estimated glomerular filtration rate; SCORE2/2-OP, Systematic COronary Risk Evaluation 2/ 2-Older Persons

Table S1. Cardiovascular risk groups based on age-specific SCORE2 thresholds

	<50 years	50–69 years	≥70 years
**Low-to-moderate CVD risk**	<2.5%	<5%	<7.5%
**High CVD risk:**	2.5 to <7.5%	5 to <10%	7.5 to <15%
**Very high CVD risk:**	≥7.5%	≥10%	≥15%

Source: ESC/EAS Guidelines on cardiovascular disease prevention in clinical practice
Abbreviations: CVD. Cardiovascular disease; ESC/EAS, European Society of Cardiology / European Atherosclerosis Society

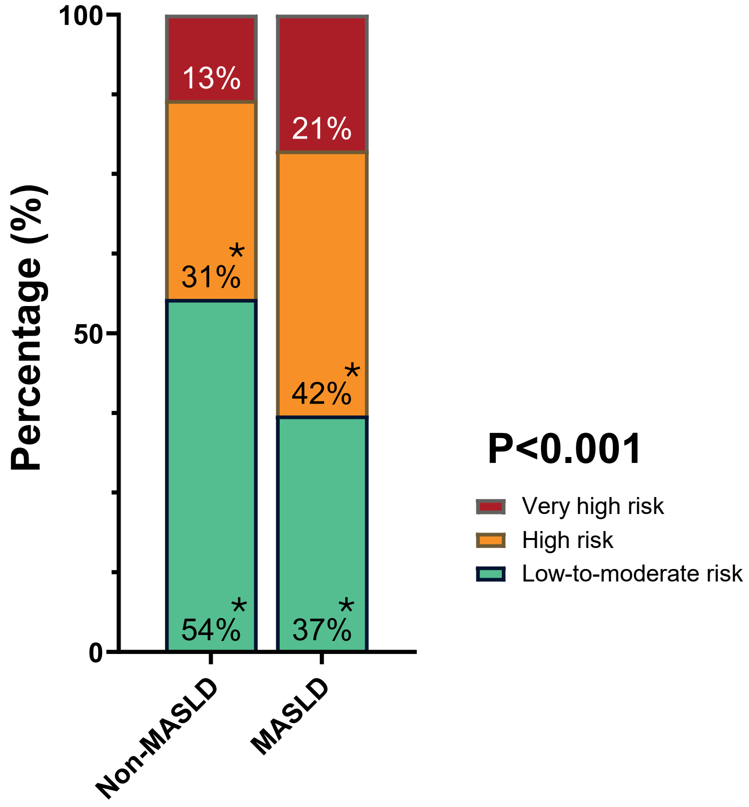

Fig. S2. Age-specific predicted 10-year CVD risk stratified for non-MASLD and MASLD participants according to the 2025 ESC/EAS guidelines on cardiovascular disease prevention. Abbreviations: CVD, cardiovascular disease; ESC/EAS, European Society of Cardiology / European Atherosclerosis Society; MASLD, metabolic dysfunction-associated steatotic liver disease. Categories are based age specific cut-offs that can be found in Supplementary table 1.

$p < 0.05$ for differences between corresponding group. . Results obtained using chi-square test.

Table S2. Likelihood of MASLD and elevated liver stiffness based on statin treatment recommendations

	N	MASLD			LSM ≥8 kPa		
		aOR	95% CI	P	aOR	95% CI	P
Statin recommended, but not used	1388		Ref			Ref	
Statin considered but not used	1986	0.80	0.67 – 0.96	0.016	0.79	0.55 – 1.13	0.199
Statin used	1308	0.74	0.61 – 0.89	0.002	0.63	0.45 – 0.89	0.009
Statin not recommended and not used	1263	0.54	0.41 – 0.71	<0.001	0.74	0.41 – 1.35	0.331

Results were obtained with logistic regression models and presented as adjusted odds ratios (aORs) with 95% CI.
Models are adjusted for age, sex, diabetes, waist circumference, current smoking, alcohol consumption (grams per day) household income (tertiles) and educational attainment.
